# Supplementary material for: COZOID: contact zone identifier for visual analysis of protein-protein interactions
Source: BMC Bioinformatics. 2018 Apr 6;19:125. doi: 10.1186/s12859-018-2113-6 (PMC5889581; doi:10.1186/s12859-018-2113-6)
Supplement: Supplementary file 2 — Software build. Executable binary file of the software tool. (ZIP 118,784 kb) [file 12859_2018_2113_MOESM2_ESM.zip › cozoid/platform/docs/testng-6.5.1-javadoc.zip!/org/testng/annotations/ITestAnnotation.html]

ITestAnnotation (TestNG)


---


|  |  |  |
| --- | --- | --- |
| **Overview** \| **Package** \| **Class** \| **Tree** \| **Deprecated** \| **Index** \| **Help** | |  |
| **PREV CLASS**   **NEXT CLASS** | **FRAMES**    **NO FRAMES**     **All Classes** |  |
| SUMMARY: NESTED \| FIELD \| CONSTR \| METHOD | DETAIL: FIELD \| CONSTR \| METHOD |  |


---


## org.testng.annotations Interface ITestAnnotation

**All Superinterfaces:**: IAnnotation, org.testng.internal.annotations.IDataProvidable, IParameterizable, ITestOrConfiguration

---

``` public interface ITestAnnotation extends ITestOrConfiguration, org.testng.internal.annotations.IDataProvidable ```

Encapsulate the @Test / @testng.test annotation.
Created on Dec 20, 2005

**Author:**
:   Cedric Beust

---

| **Method Summary** | |
| --- | --- |
| `boolean` | `getAlwaysRun()`             If set to true, this test method will always be run even if it depends on a method that failed. |
| `java.lang.String` | `getDataProvider()` |
| `java.lang.Class<?>` | `getDataProviderClass()` |
| `java.lang.Class<?>[]` | `getExpectedExceptions()` |
| `java.lang.String` | `getExpectedExceptionsMessageRegExp()` |
| `int` | `getInvocationCount()`             Returns the number of times this method should be invoked. |
| `int` | `getPriority()`             The scheduling priority. |
| `IRetryAnalyzer` | `getRetryAnalyzer()` |
| `boolean` | `getSequential()` |
| `boolean` | `getSingleThreaded()` |
| `int` | `getSuccessPercentage()`             The percentage of success expected from this method. |
| `java.lang.String` | `getSuiteName()` |
| `java.lang.String` | `getTestName()` |
| `int` | `getThreadPoolSize()`             The size of the thread pool for this method. |
| `boolean` | `ignoreMissingDependencies()` |
| `long` | `invocationTimeOut()` |
| `void` | `setAlwaysRun(boolean f)` |
| `void` | `setDataProvider(java.lang.String v)` |
| `void` | `setDataProviderClass(java.lang.Class<?> v)` |
| `void` | `setExpectedExceptions(java.lang.Class<?>[] e)` |
| `void` | `setExpectedExceptionsMessageRegExp(java.lang.String e)` |
| `void` | `setIgnoreMissingDependencies(boolean ignore)` |
| `void` | `setInvocationCount(int l)` |
| `void` | `setInvocationTimeOut(long timeOut)` |
| `void` | `setPriority(int priority)` |
| `void` | `setRetryAnalyzer(java.lang.Class<?> c)` |
| `void` | `setSequential(boolean f)` |
| `void` | `setSingleThreaded(boolean f)` |
| `void` | `setSkipFailedInvocations(boolean skip)` |
| `void` | `setSuccessPercentage(int s)` |
| `void` | `setSuiteName(java.lang.String s)` |
| `void` | `setTestName(java.lang.String s)` |
| `void` | `setThreadPoolSize(int n)` |
| `boolean` | `skipFailedInvocations()` |

**Methods inherited from interface org.testng.annotations.ITestOrConfiguration**

`getDependsOnGroups, getDependsOnMethods, getDescription, getGroups, getTimeOut, setDependsOnGroups, setDependsOnMethods, setDescription, setGroups, setTimeOut`

**Methods inherited from interface org.testng.annotations.IParameterizable**

`getEnabled, getParameters, setEnabled`

**Method Detail**

### getInvocationCount

```
int getInvocationCount()
```

:   Returns the number of times this method should be invoked.

    :   **Returns:**: the number of times this method should be invoked.

---


### setInvocationCount

```
void setInvocationCount(int l)
```

---


### getThreadPoolSize

```
int getThreadPoolSize()
```

:   The size of the thread pool for this method. The method will be invoked
    from multiple threads as specified by invocationCount.
    Note: this attribute is ignored if invocationCount is not specified

---


### setThreadPoolSize

```
void setThreadPoolSize(int n)
```

---


### getSuccessPercentage

```
int getSuccessPercentage()
```

:   The percentage of success expected from this method.

---


### setSuccessPercentage

```
void setSuccessPercentage(int s)
```

---


### getAlwaysRun

```
boolean getAlwaysRun()
```

:   If set to true, this test method will always be run even if it depends
    on a method that failed. This attribute will be ignored if this test
    doesn't depend on any method or group.

---


### setAlwaysRun

```
void setAlwaysRun(boolean f)
```

---


### getExpectedExceptions

```
java.lang.Class<?>[] getExpectedExceptions()
```

---


### setExpectedExceptions

```
void setExpectedExceptions(java.lang.Class<?>[] e)
```

---


### getExpectedExceptionsMessageRegExp

```
java.lang.String getExpectedExceptionsMessageRegExp()
```

---


### setExpectedExceptionsMessageRegExp

```
void setExpectedExceptionsMessageRegExp(java.lang.String e)
```

---


### getSuiteName

```
java.lang.String getSuiteName()
```

---


### setSuiteName

```
void setSuiteName(java.lang.String s)
```

---


### getTestName

```
java.lang.String getTestName()
```

---


### setTestName

```
void setTestName(java.lang.String s)
```

---


### getSequential

```
boolean getSequential()
```

---


### setSequential

```
void setSequential(boolean f)
```

---


### getSingleThreaded

```
boolean getSingleThreaded()
```

---


### setSingleThreaded

```
void setSingleThreaded(boolean f)
```

---


### getDataProvider

```
java.lang.String getDataProvider()
```

:   **Specified by:**: `getDataProvider` in interface `org.testng.internal.annotations.IDataProvidable`

---


### setDataProvider

```
void setDataProvider(java.lang.String v)
```

:   **Specified by:**: `setDataProvider` in interface `org.testng.internal.annotations.IDataProvidable`

---


### getDataProviderClass

```
java.lang.Class<?> getDataProviderClass()
```

:   **Specified by:**: `getDataProviderClass` in interface `org.testng.internal.annotations.IDataProvidable`

---


### setDataProviderClass

```
void setDataProviderClass(java.lang.Class<?> v)
```

:   **Specified by:**: `setDataProviderClass` in interface `org.testng.internal.annotations.IDataProvidable`

---


### getRetryAnalyzer

```
IRetryAnalyzer getRetryAnalyzer()
```

---


### setRetryAnalyzer

```
void setRetryAnalyzer(java.lang.Class<?> c)
```

---


### skipFailedInvocations

```
boolean skipFailedInvocations()
```

---


### setSkipFailedInvocations

```
void setSkipFailedInvocations(boolean skip)
```

---


### invocationTimeOut

```
long invocationTimeOut()
```

---


### setInvocationTimeOut

```
void setInvocationTimeOut(long timeOut)
```

---


### ignoreMissingDependencies

```
boolean ignoreMissingDependencies()
```

---


### setIgnoreMissingDependencies

```
void setIgnoreMissingDependencies(boolean ignore)
```

---


### getPriority

```
int getPriority()
```

:   The scheduling priority. Lower priorities get scheduled first.

---


### setPriority

```
void setPriority(int priority)
```


---


|  |  |  |
| --- | --- | --- |
| **Overview** \| **Package** \| **Class** \| **Tree** \| **Deprecated** \| **Index** \| **Help** | |  |
| **PREV CLASS**   **NEXT CLASS** | **FRAMES**    **NO FRAMES**     **All Classes** |  |
| SUMMARY: NESTED \| FIELD \| CONSTR \| METHOD | DETAIL: FIELD \| CONSTR \| METHOD |  |


---
